# Collaborative noise data collected from smartphones

**DOI:** 10.1016/j.dib.2017.07.039

**Published:** 2017-07-21

**Authors:** Erwan Bocher, Gwendall Petit, Judicaël Picaut, Nicolas Fortin, Gwenaël Guillaume

**Affiliations:** a CNRS, Lab-STICC Laboratory UMR 6285, Vannes, France; b Université de Bretagne Sud, Lab-STICC Laboratory UMR 6285, Vannes, France; c IFSTTAR, AME, LAE, F-44344 Bouguenais, France; d Cerema, Direction Territoriale Est, Groupe Acoustique, Strasbourg, France

**Keywords:** Noise, GIS, SDI, OGC, Crowdsourcing, VGI, Smartphones

## Abstract

Noise stands for an important human health and environmental issue. Indeed, noise causes annoyance and fatigue, interferes with communication and sleep, damages hearing and entails cardiovascular problems (WHO, 2011) [1]. From an environmental point of view, noise implies a lessening of both the richness and abundance of the animal species, an alteration of the communication, which can threaten the reproduction and predation, etc. (Newport et al., 2014; Shannon et al., 2014) [2], [3]. Consequently, effects related to environmental noise result in a huge cost for society, with 2.2 billion euros in France, for example, for the year 2013 (Bourges and Diel, 2015) [4]. In this context, the reduction of noise in the environment is a burning issue, which requires, firstly, carrying out an evaluation of noise in the environment, and secondly, to establish action plans to reduce noise annoyance. With the development of the concept of participatory measurement, and considering the extremely large number of people equipped with a smartphone while being "in mobility", the use of smartphones is potentially a relevant solution to realize a large-scale environmental noise evaluation.

The data presented hereinafter are collected from the Android NoiseCapture application and shared from the OnoMap Spatial Data Infrastructure (SDI). The NoiseCapture approach consists in measuring noise along a path, and then to share data with the community. This approach has been developed within the framework of the European ENERGIC-OD^1^ project, which aims at deploying a set of Virtual Hubs (VH) to share heterogeneous data with third parties, in respect with the European INSPIRE, and at developing new and original services that can be useful for the community.

The noise data that are acquired by volunteers around the world (citizen observations), are organized in three files, containing the path of measures (a set of points), standardized noise indicators, noise description and other useful variables (GPS accuracy, speed…). These data can be very relevant later to propose an environmental noise evaluation, through simple or complex treatments.

**Specifications Table**

**Table:** 

Subject area	*Environmental science*
More specific subject area	*Environmental Noise, Geography, Acoustics*
Type of data	*Zipped files including GeoJSON files*
How data was acquired	*The data are collected from the NoiseCapture*[5]*application and offered by the OnoMap*[6]*processing services.*
Data format	*Raw and computed.*
Experimental factors	*Noise indicators are calculated from audio recording*
Experimental features	*Combination of acoustical techniques and spatial analysis methods in a standardized open geospatial framework*
Data source location	*The data are available all around the world. The spatial coverage depends on the community contributions.*
Data accessibility	*The data are available as open data license and daily updated on the website*http://data.noise-planet.org/noisecapture/

**Value of the data**

• Noise data are computed according to standardized procedures in order to propose classical noise indicators, such as the equivalent sound level LAeq [5]. Such data are consistent with the European Directive 2002/49/EC (noise regulation), and can be used to produced useful analysis of the noise environment.
• With the concept of participatory measurements, and considering the extremely large number of people equipped with a smartphone, the propose approach potentially provides access to an extremely large amount of data, which is otherwise homogeneous throughout the world.
• Data can be used by scientists, acousticians or experts, in order to build new data analysis methods (spatio-temporal, geostatistics) and data visualization tools (real time filtering, dashboard, map styling), in order to evaluate and represent the environmental noise.
• Because additional information can be provided by the user to describe its own perception of the noise environment, through “tags” and a personal evaluation of the pleasant or unpleasant nature of noise, these data offers the opportunity to better correlate noise perception and physical noise indicators.

## 1 Data

The noise measurements collected from smartphones are organized in three zipped GeoJSON files. All GeoJSON files are based on the EPSG 4326 coordinate reference system^2^. A GeoJSON file is prefixed with the name of the country and its administrative divisions, replaced bellow by the * symbol.

### 1.1 *.tracks.geojson

This file contains the data collecting along a GPS track. A GPS track is defined by 8 property values. Note that each feature of the GeoJSON is a measurement session over a time period (expressed in seconds). The geometry corresponds to the bounding box of measurements locations.

• time_ISO8601: start time of measurement, using the ISO 8601 representation.
• time_epoch: start time of measurement in Unix Time Stamp (UTS) format (number of milliseconds since January 1st, 1970, 00:00:00 GMT). The timezone is provided by the localization of the measure, not by the configuration of the smartphone.
• pk_track: Database of track primary keys. This value may change when the zip files are updated, but are unique in the same zip file. This value is linked with the *.points.geojson file and could be used to build a geometry representation of the path of the measures.
• track_uuid: Track Universally Unique Identifier. This unique value never changes between zip updates.
• gain_calibration: Signal gain in dB, provided by the user after the noise calibration of the smartphone. Indeed, it is recommended to the user to proceed to an acoustic calibration of its smartphone, using the specific feature provided in the NoiseCapture application. This signal gain is already applied to all noise levels related to this track.
• noise_level: Sound level LAeq along the track.
• tags: User supplied tags for describing the noise environment along the track.
• pleasantness: User supplied pleasantness 0–100 (may be null) of the noise environment along the track.

### 1.2 *.points.geojson

This file stores the measurement points. Each feature corresponds to a measurement at each second. The file contains a geometry field called the_geom and 9 property values.

• the_geom: a point with *X*, *Y* and *Z* coordinates to store the GPS location.
• pk_track: Track primary key. This value may change when the zip files are updated, but are unique in the same zip file. This value is linked with the *.tracks.geojson file and could be used to build a geometry representation of the path of the measures.
• time_ISO8601: Time of the measurement using the ISO 8601 time representation.
• time_epoch: Time of measurement in UTS format.
• time_gps_ISO8601: GPS localisation may not be obtained each second, so the provided location is attached to the following "gps fix" time.
• time_gps_epoch: "GPS fix" time in UTS format.
• noise_level: Equivalent Noise level LAeq in dB(A), measured over a time period of 1 s.
• speed: GPS provided speed (may be not accurate).
• orientation: GPS provided orientation (may be not accurate).
• accuracy: GPS localization accuracy in meters.

### 1.3 *.areas.geojson

This file corresponds to a basic post-processing of all measurements produced by the community. All data are aggregated in hexagons, in order to produce mean noise indicators and information in each hexagon (mean LAeq, LA50, mean pleasantness) Each feature is an area represented by an hexagon of 15 m radius where all *.points.geojson have been aggregated. 11 property values are available.

• cell_q: Hexagon q coordinate using EPSG:3857 -- WGS84 Web Mercator.
• cell_r: Hexagon r coordinate using EPSG:3857 -- WGS84 Web Mercator.
• LA50: Median sound level in dB(A) (*i.e.* the noise level just exceeded for 50% of the measurement period).
• laeq: Mean equivalent sound level LAeq in dB(A).
• mean_pleasantness: Mean pleasantness 0–100 (may be null).
• measure_count: Number of measurements (equivalent to the whole measurement time in seconds) in the hexagon (may be parts of different tracks).
• first_measure_ISO_8601: Date of the first measurement in the hexagon, using ISO8601 time representation. The timezone is provided by the localisation of the measurement, not by the configuration of the smartphone.
• first_measure_epoch: Date of the first measurement in the hexagon, in UTS format.
• last_measure_ISO_8601: Date of the last measurement in the hexagon, using ISO8601 time representation. The timezone is provided by the localization of the measure, not the configuration of the smartphone.
• last_measure_epoch: Date of the last measurement in UTS.

## 2 Experimental design, materials and methods

**Fig. 1:**
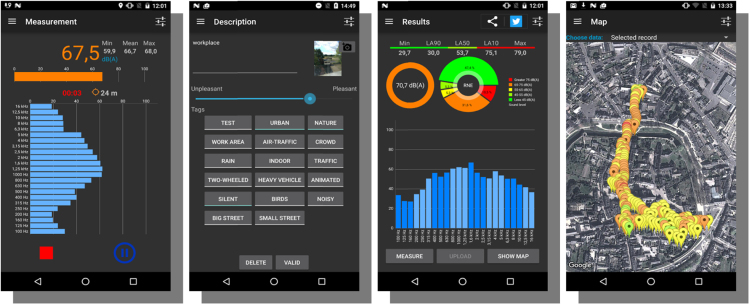
Screenshots of the NoiseCapture application (from left to right: measurement / perception / acoustic indicators / map).

A volunteer moves along a given path and performs a continuous acoustic measurement using the NoiseCapture application (Fig. 1). Audio data are then processed by the smartphone to produce results in a form of acoustic indicators (equivalent noise level LAeq, percentile indicators (LA10, LA50, LA90), min and max values of the sound level…). The user can also complete the measurement with information about his own perception of the sound environment, using tags and an information of pleasantness. Some data are then uploaded to a geoserver in order to process data and to build a noise map that merge all community results (community data are aggregated in hexagonal areas in order to produce a basic noise maps). This operation is done thanks to the Web Processing Service. It must be note that only the LAeq data are transferred to the geoserver, since all other parameters can be computed from this indicator. In addition, it is important to mention that only anonymous data are considered; there is no audio recording nor any information that could be used to identified or localized people (see the privacy policy^3^ of NoiseCapture).

**Fig. 2:**
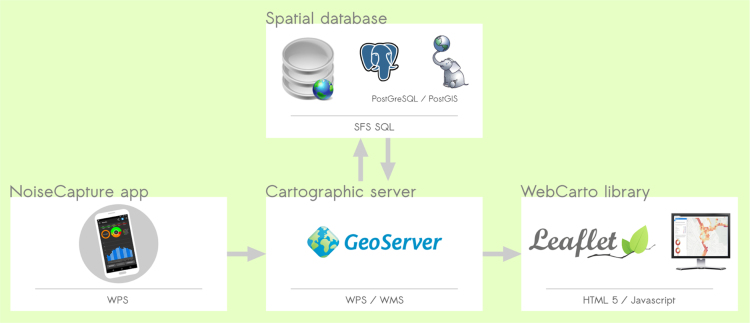
OnoMap architecture and tools.

The server side services are organized around a web geoservices application and a relational database management system (Fig. 2). The geoservice application uses the open source GeoServer software and a plugin to support the Web Processing Service (WPS) standard. The WPS standard is used to execute 3 chained processing (scripts).

• *Process 1: Push the data from the smartphone*. A WPS is used to push a zipped file that contains the measurements, from the smartphone to the PostGIS relational database. The zipped file is unzipped on the server and data are inserted into the database.
• *Process 2: Update the hexagon maps*. When the data is uploaded into the database, a second WPS script starts to build the hexagon and compute the mean noise indicators and information.
• *Process 3: Export the data in GeoJSON files*. A daily script is executed to export the noise measures for a specific country (and administration divisions) from the PostGIS database to a zipped folder made of the 3 GeoJSON files, as detailed above.

The measures stored in the PostGIS database are dynamically used to create thematic maps thanks to the Web Map Service specifications (WPS). Three WMS layers are rendered in a web application available at http://noise-planet.org/en/map.html.

## Supplementary material

Because OnoM@p and NoiseCapture tools are open source software, all codes or resources are available on the GitHub repository: https://github.com/Ifsttar/NoiseCapture. In the meantime, the NoiseCapture data are freely available at http://data.noise-planet.org/en/index.html.

## Footnotes

2 http://spatialreference.org/ref/epsg/wgs-84/

3 http://noise-planet.org/en/NoiseCapture_privacy_policy_history.html
